# Esophageal Actinomycosis in an Immunocompetent Patient Mimicking Carcinoma: A Case Report

**DOI:** 10.31729/jnma.7354

**Published:** 2022-06-30

**Authors:** Shekhar Poudel, Abhin Sapkota, Rahul Devkota, Sujan Chandra Poudel, Angel Dangol

**Affiliations:** 1 Department of Gastroenterology, Kathmandu Medical College Teaching Hospital, Sinamangal, Kathmandu, Nepal; 2 Vayodha Hospitals Private Limited, Balkhu, Kathmandu, Nepal

**Keywords:** *actinomycosis*, *carcinoma*, *case reports*, *esophagus*

## Abstract

Esophageal actinomycosis is a rare occurrence that presents a diagnostic challenge due to its vague clinical picture. The common symptoms include dysphagia, odynophagia and epigastric pain. These symptoms, although alarming, are usually non-specific. In this report, we describe an immunocompetent 38-year-old woman who presented with dysphagia and burning chest pain. Her initial examination and investigations suggested carcinoma of the oesophagus. On further evaluation and histopathology examination, she was diagnosed with esophageal actinomycosis and managed with antibiotics and symptomatic relief. She had significant improvement on follow up examination. The diagnosis of this condition in an immunocompetent patient can be confusing and requires a high degree of suspicion.

## INTRODUCTION

Esophageal actinomycosis is a rare infection of the oesophagus generally only seen in immunocompromised individuals. Occurrence in immunocompetent patients without predisposing risk factors is exceedingly rare. Less than 30 cases of esophageal actinomycosis have been reported in the literature.^1^ It presents as a granulomatous infection with features of esophageal inflammation or ulceration manifesting as dysphagia, odynophagia and epigastric pain.^2^ The presence of vague overlapping symptoms often causes a diagnostic dilemma. Here, we present a case of a 38-year old immunocompetent female who had a history of gradual onset dysphagia and burning pain over the central chest with esophageal actinomycosis mimicking a carcinoma.

## CASE REPORT

A 38-year-old woman presented to our hospital with a 2 months history of gradual onset dysphagia for solid food more than liquid food. She also had moderate intensity burning pain over the central chest, only present when swallowing food, with no radiation or postural variation but was associated with weight loss. She initially tried managing the pain and discomfort at home with over the counter medications. Lately, her dysphagia had been worsening for which she presented to the outpatient department. There was no significant medical or family history.

She had no history of fever, dyspnea, vomiting, acid reflux or malena. The patient denied tobacco use or excessive consumption of alcohol. On initial examination, she only had mild epigastric discomfort with no additional significant findings. She had normal vitals and her Electrocardiogram (ECG) was unremarkable. Laboratory investigations showed a normal complete blood count, normal biochemistry panel, and negative serology for Human Immunodeficiency Virus (HIV). She also tested negative for Coronavirus Disease (COVID-19) and was admitted for further evaluation in the general ward. She was initially managed symptomatically.

**Figure 1:**
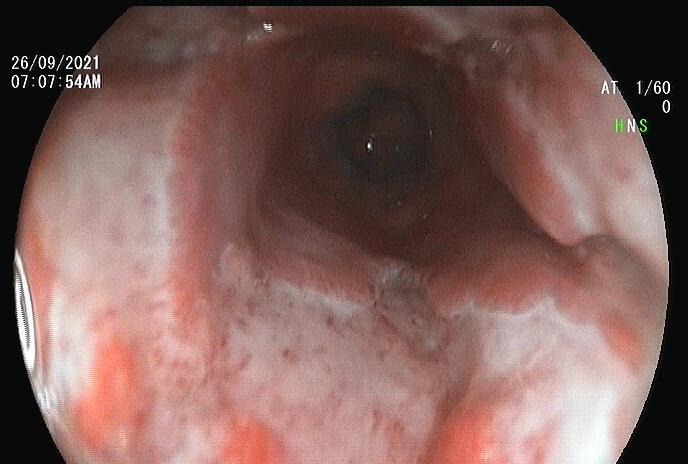
Endoscopy showing friable ulcero-proliferative lesions in the distal oesophagus.

Ultrasound examination of the abdomen and pelvis showed normal findings. A Contrast-enhanced Computed Tomography (CECT) scan of the chest was done which showed asymmetrical circumferential wall thickening noted in the lower oesophagus with a sub centimetre sized surrounding lymph node with suspicion of carcinoma so a follow-up endoscopy guided biopsy advised. An Upper Gastrointestinal (UGI) endoscopy showed white plaques predominantly in the mid-oesophagus with multiple 1-2 cm shallow ulcero-proliferative lesions and a 3x2 cm, irregular, malignant-appearing ulcer in the distal oesophagus (Figure 1).

**Figure 2:**
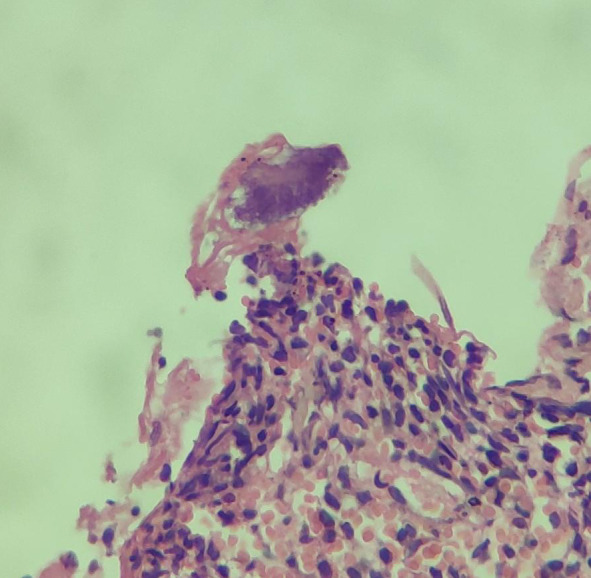
Biopsy of the distal oesophageal lesion showing sulphur granule.

Histological examination of biopsies taken from the oesophagus revealed esophageal ulceration defect with reparative nuclear atypia and infection by *Actinomyces* species suggested by the presence of sulphur granules (Figure 2).

No dysplasia, malignancy, fungi, or viral inclusions were present. A diagnosis of esophageal actinomycosiswas made and intravenous (IV) ceftriaxone 1 gm twice daily was given for 7 days followed by oral amoxicillin 500 mg which was administered for 2 weeks along with other supportive treatment. The patient improved clinically following medical treatment. At 10 weeks follow up, she had improved symptoms and all examination findings were normal. A repeat UGI endoscopy showed marked healing of the shallow ulcers, reduced size of the malignant-appearing ulcer, and improvement in the surrounding inflammation.

## DISCUSSION

*Actinomyces* are gram-positive, facultative anaerobic human commensal bacteria generally found in the oral cavity and Gastrointestinal (GI) tract. In the GI tract, the most common regions affected by actinomycosis are the cecum, appendix and colon.^3^ Esophageal actinomycosis is a very rare occurrence, especially in those that are immunocompetent without any underlying risk factors. Actinomycosis infections are more commonly seen in the male population but our patient was an immunocompetent female without any known risk factors.^4^

*Actinomyces* infections usually have a history of preceding mucosal injury resulting from surgery, trauma or instrumentation. Impaired mucosal defences in the background of an immunocompromised state provide a suitable environment for infections.^5^ Our patient had no such history of mucosal trauma due to any known cause. This accompanied by her healthy immune system made her an unlikely candidate for EA. We assumed that some unknown event like food-induced trauma or gastroesophageal reflux may have played some role in our patient.

The diagnosis of esophageal actinomycosis presents a challenging scenario as the symptoms are vague and often confused with other conditions or even other infections causing esophagitis. Furthermore, the findings on radiology are very non-specific. Computed Tomography (CT) scan may show a thickening of the esophageal walls during the early phase of infections with occasional development of sinus tracts as the infection progresses.^6^ In our case, we also had similar findings of lower esophageal wall thickening. This coupled with the presence of multiple subcentimeter lymph node enlargements led us to think of possible malignant aetiology.

A case of esophageal actinomycosis masquerading as cancer was reported in a 46-year old woman presented on UGI endoscopy as an esophageal mass.^7^ In another case of a 27-year-old male with features of esophagitis diagnosed as OA, UGI endoscopy showed an ulcerated lesion.^8^ In our case, malignant looking ulceration was seen which is usually the more common presentation of esophageal actinomycosis on UGI endoscopy.^9^

*Actinomyces* are isolated and identified only in a few cases as it is very difficult to culture this bacteria due to a variety of reasons like inadequate sampling, crosscontamination or prior antibiotic therapy.^7^ So, microscopy plays a crucial role in diagnosis, which usually presents as necrotic tissue, fungal-like Gram-positive filamentous organisms and the characteristic yellowish coloured "sulphur granules".^3^ Histopathological examination of the biopsy specimen in our case also showed the presence of Sulphur granules which guided us to the correct diagnosis.

The recommended regimen for the treatment of abdominal forms and/or thoracic actinomycosis is 4-6 weeks of Intravenous (IV) penicillin, 3-4 million units intravenously every 4 hours, followed by oral penicillin or amoxicillin for 6-12 months.^10^ For those with penicillin allergy tetracycline is preferred. Other viable alternatives include ceftriaxone, clindamycin, minocycline and imipenem.^11^ In our case we started with IV ceftriaxone 1 gm initially followed by oral amoxicillin 500 mg along with medication for symptomatic relief. At 10 weeks follow up she had marked improvement in symptoms and a repeat UGI endoscopy also showed significant healing of previous lesions. She was satisfied with the treatment she received and was able to go back to her daily activities.

Esophageal actinomycosis as a cause of oesophagitis remains a rare and challenging clinical scenario in gastroenterology. A limited number of cases of esophageal actinomycosishave been reported with only a handful of those occurring in immunocompetent individuals. Due to its non-specific features which can easily be confused with malignancy, it is likely to be misdiagnosed. Physicians should be aware of esophageal actinomycosis as a possible cause of dysphagia. It requires adequate clinical knowledge and a high degree of suspicion to diagnose OA, especially in immunocompetent individuals.
